# Description of the Resin Curing Process—Formulation and Optimization

**DOI:** 10.3390/polym11010127

**Published:** 2019-01-12

**Authors:** Aleksander Muc, Paweł Romanowicz, Małgorzata Chwał

**Affiliations:** Institute of Machine Design, Cracow University of Technology, ul. Warszawska 24, 31-155 Kraków, Poland; promek@mech.pk.edu.pl (P.R.); mchwal@pk.edu.pl (M.C.)

**Keywords:** fundamental relations of resin flow and hardening, residual stresses, thermosetting resin curing process, optimization, design variables

## Abstract

The paper gives a set of basic relations characterizing the phenomena of viscous polymer resin flow through fiber reinforcement and the resin curing process. We describe the technological process of manufacturing composite structures. The influence of the resin curing process on values of residual stresses in composite constructions is analyzed taking into account two components: thermal shrinkage and chemical shrinkage of resins. For cases of 2-D structures, the method of formulating such tasks has been demonstrated. The types of design variables appearing in the optimization problems in this area are also presented. The 2-D optimization problems have been formulated. Various optimization problems are solved in order to demonstrate the influence of discussed relations on values of residual stresses and curing processes of thermosetting resins.

## 1. Introduction

The process of producing finished products of fiber composites is based on matrix polymerization. The problems related to the kinetics of reactions (degradation and polymerization), physical changes and the mechanical properties of polymers and monomers are studied in the field of science known as polymer physics. Carrothers [1] is the first person who analyzed the in step-growth polymerization of monomers. However, Flory [2] is considered the first scientist establishing the field of polymer physics. French scientists [3] and Russian/Soviet schools of physics contributed much since the 70s [4,5]. However, the development and achievements of polymer physics seem to be not sufficient in the description of the fabrication of constructions made of fibre composites since they are produced in two phases, i.e., polymers and reinforcement. The final products can be created simultaneously with the creation of the material, which is generally the case of thermosetting resin matrix composites. In thermoplastic resin composites, it is more common to fabricate the composite first and form or mold the shape in the second operation [6,7,8,9].

Starting materials of a thermosetting resin are in a fluid state and are called monomers or prepolymers. They are solidified by a chemical reaction during which molecules of monomers or prepolymers are linked together to form polymer networks [10]. This process of linking the molecules is called polymerization or cross-linking. The cross-linking is accomplished by catalysts or curing agents usually selected to give the desired combination of time and temperature to complete the reaction suitable for a particular product. The curing and accompanying hardening of thermosetting polymers are irreversible. The curing can be accomplished in stages. The composite can be formed in one stage when polymer viscosity is low for good penetration into fiber bundles, and the final curing and hardening carried out when the product is shaped. The interfacial bonding between two polymers can be controlled using a straightforward and direct method [11].

**Figure 1:**
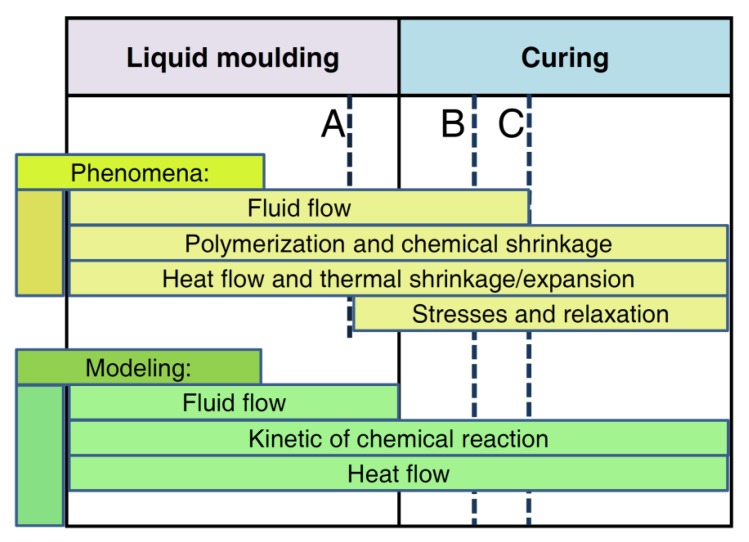
Phenomena occurring in the technological process related to the curing of resins: A—exceeding the gelling threshold, B—curing the area near the infusion channel (blockage of the infusion), C—resin cured in the entire volume.

The hardening process (in the case of thermosetting-based composites) or solidification from melt/fluid state (for thermoplastic-based composites) is an important part of the technological process of manufacturing of advanced composite structures. A comprehensive discussion of these processes in composite materials would include many complex phenomena such as mass, momentum and heat transfer, accompanied by the chemical curing reaction of the resin as well as motion and deformation of fibers (Figure 1). Hardening/solidification processes are usually carried out with the use of the pressure, which leads to the squeezing air and resin out of the structure. Such transformations result in changes in the microstructure and dimensions of the composite materials. These techniques have been used in the manufacturing of composite parts; however, the hardening process has more crucial steps in the fabrication of thermosetting-based composite structures. Controlling and proper management of these important processing steps allow achieving the required properties of composite materials, especially those that are dominated by the fibers. Improper hardening/solidification can lead to the unacceptable structural defects such as residual stresses, warping, voids or other unwanted effects. In many cases, the presence of such defects may result in the rejection of the structure.

A number of various factors that are not fully recognized and defined plays a fundamental role here. The most important of them are given below:

- knowledge about the size, physicochemical structure and interfacial surface form, which in many cases is the source of macro-cracks and the final damage of the structure,
- description of the resin curing relationship through a series of semi-empirical relations containing many material constants that are very difficult to determine in the experimental path; moreover, little information on this subject can be found in the generally available literature,
- lack of simple relations describing the deformations of the viscoelastic matrix as a function of resin hardening parameters and temperature,
- phenomenological form of the infiltration relations of the resin into the fiber bundles, which is especially complicated for 3-D cases,
- the need to analyze complex, non-linear physical relations for various types of initial-boundary conditions, which is sometimes very complicated.

Due to the above facts, it is necessary to conduct numerical analysis using the finite element method (FEM) or the finite volume method (FVM), and then validate numerical results with experiments and engineering experience [12,13,14]. Performing numerical calculations already allows for some optimization of the technological process by eliminating the most unfavorable solutions, even without precisely specifying particular objective functions. Unfortunately, the basic inconvenience of accurate numerical analysis is their time-consumption, which increases dramatically in the case of optimization [15,16,17,18].

Considerations and analysis in this area can not only be limited to the description of the resin curing process but must also be supplemented with a description of the mold pouring/filling process and infiltration through the fiber system. A detailed description of the physicochemical phenomena associated with the process of manufacturing composite structures are shown in Figure 1, which also presents what phenomena can be easily modeled using the finite element/volume method (part referred to as simulation). Others such as chemical shrinkage, residual stresses, etc., require the development of additional models.

In the process of optimization of manufacturing technologies of composite structures, the viscosity [19,20,21] and the reaction kinetic models are used. Application of them allows for the determination of the optimal shape of a mold and the optimal process of heating and cooling of the mold and sample (a temperature-time history, a location of inlet gates and a location of heaters). Many different reaction kinetics (RK) models have been proposed so far [22,23,24,25,26,27,28,29,30,31,32,33,34,35,36,37]. In practical application, the most often used are models (or their extended or developed formulations) proposed by Kamal and Sourour [22] and Bogetti and Gillespie [23].

The above-mentioned models have been successfully applied to the different composite materials and resin systems such as carbon-fiber reinforced polymer (CFRP) [38,39,40,41,42,43,44], glass-fiber reinforced polymer (GFRP) [45,46,47,48], epoxy resin systems [49,50,51], fast cure epoxy [30,32,37]. The applicability of the curing models has been also confirmed for different manufacturing technologies of composite structures, such as: liquid silicone rubber (LSR) [52], resin film infusion (RFI) [53], resin transfer molding (RTM) [30,31,33,38,44,51,54], compression resin transfer molding (C-RTM) [32], reactive injection molding (RIM) [30], vacuum-assisted resin transfer molding (VARTM) [55,56], reactive extrusion (REX) [30], autoclaving [57] and out-of-autoclave (OOA) [34,37]. A detailed description is presented in Chapter 2.

An optimal cure process of composite structures was proposed by White and Hahn [58,59] to decrease residual stresses during the manufacturing process. Muc [12] and Muc and Saj [15,16] conducted research on the optimal design of composite thermoforming using a genetic algorithm. Matysiak et al. [52] optimized the silicone molding process based on numerical simulations and experiments. Song et al. [60] presented optimization of the curing procedure to minimalize the residual voids. Leite et al. [61] applied artificial neural networks to optimize the vacuum thermoforming process and minimalize the product deviations.

The present paper begins with the description of the basic relations describing the phenomena occurring throughout the manufacturing process of composite structures. In this section, the flow of the fluid (resin), relations of heat flow during mold heating, kinetic model of exothermic chemical reaction, the viscosity of the resin during its curing, and resin infiltration through reinforcement are put forward. In the next chapter, the residual stresses arising in composite material manufacturing are presented and discussed with the use of the viscoelastic and the mechanical 2D models. The optimization problems are shown in the following section, in which the influence of the resin curing process on values of residual stresses in composite construction is analyzed taking into account two components: thermal shrinkage and chemical shrinkage of resins. For cases of 2-D structures, two particular technological processes are presented. In these examples, the influence of outside heater temperature on the distribution of the degree of cure has been demonstrated. In Appendix A, the physical properties of the epoxy resin used in the numerical examples are given.

The aim of the present paper is two-fold:

1. to demonstrate the influence of the fundamental relations and models on the values of residual stresses,
2. to formulate and solve possible optimization problems arising in the curing of thermosetting resins.

The influence of the fundamental relations and the resin curing models (described in chapters 2 and 3) on the values of the residual stresses is presented and discussed in paragraph 3.4. It was observed that the number of assumed curing variables (and their dependence) in the curing model have a significant influence on the calculated residual stresses.

## 2. Relations Used to Describe the Phenomenon of Curing

The hardening process of resins is a complex physicochemical phenomenon, for which description a number of relations are required—see also Figure 1:

- description of the motion of the fluid (resin) into the form of a product,
- relations of heat flow during mold heating by external heat sources,
- kinetic model of exothermic chemical reaction,
- description of the law of viscosity of the resin during its curing,
- modeling phenomena of resin infiltration through reinforcement.

Each of the above physical laws includes a number of material factors, which, in many cases are very difficult to determine.

To describe the motion of the fluid, we apply non-linear relations for viscous liquids in the form proposed by Navier-Stokes—see Landau et al. [62]. The system of equations will be formulated in the Euler approach (description at a fixed point in space), which is directly related to the numerical method used to solve equations (finite volumes). Fundamental relations are derived from a series of conservation laws listed below:

the principle of mass conservation (equation of flow continuity):

(1) $$\frac{d\rho }{dt}+\rho \nabla \cdot v=0,$$

the second law of Newton’s dynamics (equation of fluid motion):

(2) $$\rho \left(\frac{\partial v}{\partial t}+v\nabla \cdot v\right)=\rho F+\nabla \cdot Τ,$$

the principle of energy conservation:

(3) $$\rho c_{v}\frac{dT}{dt}=T:\nabla v+\dot{\sigma }+\nabla \cdot \dot{q},$$

where ***T*** is the stress tensor in the liquid and it is the sum of the hydrostatic pressure *p* and the viscous friction:

(4) $$T=-pI+\frac{2}{3}\eta \nabla \cdot vI+\eta \left(\frac{\partial v_{i}}{\partial x_{j}}+\frac{\partial v_{j}}{\partial x_{i}}\right),$$

and *v* is the speed vector having the component *v*_i_, ρ is the density of the fluid, ***I***—the unit matrix, *c*_v_—the specific heat in a constant volume. d*H*/d*t* = ∂*H*/∂*t* + ***v***∇*H*—material derivative of variable *H*, the symbol “:” means double scalar product, and the symbol ∇ defines a gradient.

For most of the continuous media, it is assumed that the heat flux vector $\dot{q}$ is proportional to the temperature gradient. This relation is called the Fourier law:

(5) $$\dot{q}=\lambda \nabla T,$$

where: *T*—temperature, *λ*—thermal conduction coefficient.

Here, for thermosetting, the viscosity model proposed by Castro-Macosko [19] is used:

(6) $$\eta =B\exp\left(\frac{T_{b}}{T}\right){\left(\frac{\alpha_{g}}{\alpha_{g}-\alpha }\right)}^{C_{1}+C_{2}\alpha },$$

where: *B*—reference viscosity [Pas], *T_b_* = *E*/*R*—parameter dependent on the activation energy *E* [K], *C*_1_, *C*_2_—coefficients, *R*—gas constant = 8.314 J/(mol·K). The flow threshold α_g_ (generally for epoxy-amine systems α_g_ = 0.5 − 0.6 [20]) is synonymous with gelling of the resin, i.e., the moment in which the material rapidly changes from a liquid state to a solid state—highly elastic. This is mainly due to the rapid increase of the viscosity of the material.

The degree of cure (α_g_) at which gelation occurs can be calculated by formula proposed by Castro-Macosko:

(7) $$\alpha_{g}={\left[r(f-1)(g-1)\right]}^{-\frac{1}{2}},$$

where: *r*—the ratio between resin and hardener, *f* and *g*—functionality of resin and hardener, respectively. Application of the Castro-Macosko model—Equation (6), with exponential viscosity growth behavior, provides a good description of the isothermal viscosity rise [21].

The curing reaction is exothermic, which is taken into account in the equation of energy balance by determining the intensity of the internal heat source, defined by the formula:

(8) $$\dot{\sigma }=\rho Q_{T}\frac{d\alpha }{dt},$$

where *Q_T_* is experimentally determined by the total heat of the chemical reaction separated from the mass unit after the time when the material has been fully cured.

The reaction rate expressed as a derivative of the degree of curing α is calculated using the reaction kinetics (RK) model. The phenomenon of cross-linking of curable material consisting of the linking of polymer molecules chains into ordered spatial networks is a process associated with the release of large amounts of heat (the exothermic character of the phenomenon). The analysis of this process requires knowledge and understanding of the kinetics governing the reaction of cross-linking. It is difficult to consider this phenomenon at the microscopic level, i.e., to study and describe the role of individual molecules or chains. The ideal model of reaction kinetics depends on the analysis in which it will be used. If the model is to show the process, it should be relatively simple so that it can be implemented into the mathematical apparatus. If, however, there is a need to track individual components of the reaction, a kinetic model should be used that gives such possibilities. The size denoted as α is a measure of the degree of hardening (curing) of the curable material. It characterizes the state of development of the polymerization reaction by expressing the stage of reacting the curable material in the considered moment of time. Generally, the RK models can be described by the following formula [7]:

(9) $$\frac{\partial \alpha }{\partial t}=Kf\left(\alpha \right),$$

where *f*(α) is the function describing the cure rate and *K* is the pre-exponential factor.

Due to many different resin systems used in practical applications, different RK models have been proposed so far [7,22,23,24,25,26,27,28,29,30,31,32,33,34,35,36]. Many RK models are based on the following formulations (including a combination of them):

the n-th order reaction model

(10) $$f\left(\alpha \right)={\left(1-\alpha \right)}^{n},$$

the n-th order autocatalytic reaction model

(11) $$f\left(\alpha \right)={\left(1-\alpha \right)}^{n}\left(1+k\alpha \right),$$

the Prout-Tompkins autocatalytic reaction model

(12) $$f\left(\alpha \right)={\left(1-\alpha \right)}^{n}\alpha^{m}.$$

The most often used in practical applications is the model proposed by Kamal and Sourour in 1973 [22]. The model allows for determining the rate of curing reaction depending on the degree of curing α:

(13) $$\frac{\partial \alpha }{\partial t}=\left(k_{1}+k_{2}\alpha^{m}\right){\left(1-\alpha \right)}^{n},$$

The reaction rate constants *k*_1_ and *k*_2_ are usually determined from the Arrhenius relationship defined by the formula:

(14) $$k_{i}=A_{i}\exp\left(\frac{-E_{i}}{T}\right),$$

where: *i* = 1, 2; *m*, *n*—constants, *A_i_*—exponential factors [1/s], *E_i_*—activation energies [K], *T*—absolute temperature [K]. The sum of the parameters *m* + *n* defines the so-called order of reaction speed. In some versions of the model, you can find activation energy in the dimension [J/mol]. It is then necessary to further divide this amount by the constant *R* (constant gas). It should be noted that these RK models are formal ones, and hence, formally capture the actual chemical reactions.

Bogetti and Gillespie [23] stated that Kamal’s model is the most adequate for the description of phenomena occurring in the technological processes of composite structures production. It is a special case of Equation (13), in which it is assumed that *k*_1_ = 0, i.e.,:

(15) $$\frac{\partial \alpha }{\partial t}=k_{2}\alpha^{m}{\left(1-\alpha \right)}^{n},$$

There are also many simplified relations received on the basis of experimental studies. An example of such a relationship is the relation proposed by Kempner et al. [24], used to describe the curing process of the carbon fiber reinforced AS4/350-6 pre-impregnates:

(16) $$\frac{\partial \alpha }{\partial t}=\{\begin{array}{lll} \left(k_{1}+k_{2}\alpha \right)\left(1-\alpha \right)\left(B-\alpha \right) & \mathrm{for} & \alpha \leq 0.3\\ k_{3}\left(1-\alpha \right)\; & \mathrm{for} & \alpha >0.3 \end{array},$$

where *k_i_* is defined as before (Equation (14)) and *B* is a constant (for AS4/350-6 pre-impregnates *B* = 0.47).

The use of different laws describing changes in the degree of resin curing (i.e., Equations (13), (15) or (16)) leads to their different variations in time and finally to different residual stress values—see Muc [12,63,64]. Due to this fact, a proper selection of the RK model is an important practical issue. On the basis of the validation of the particular models to the experimental tests [27,28,29,30,31,32,33,34,35,36,37,38,39,40,41,42,43,44,45,46,47,48,49,50,51,52,53,54,55,56,57], the practical recommendations regarding the selection of the RK models for composite materials (Table 1) and fabrication processes of fiber composites (Table 2) are given.

For the production of composite structures, in technological processes, the liquid resin is pressed into the mold cavity, in which there is reinforcement with a defined permeability. Such a system significantly modifies the flow of the material (resin) by fibrous reinforcement. In order to characterize such a flow, Darcy’s law may be used (it is understood as the right to filter fluid through a medium with a defined permeability) in the form of:

(17) $$v=-\frac{1}{\eta }K\cdot \nabla p.$$

Tensor ***K*** is characterized by the permeability of reinforcement during the flow of resin in the liquid state and takes the following form:

(18) $$K=\left[\begin{array}{ccc} k_{11} & k_{12} & k_{13}\\ k_{12} & k_{22} & k_{23}\\ k_{13} & k_{23} & k_{33} \end{array}\right].$$

In the system of Equations (1)–(18), the set of unknowns consists of: ***v***—velocity vector with three components, ***T***—symmetrical stress tensor with six independent components, *T*—temperature, $\dot{\sigma }$—intensity of the internal heat source, $\dot{q}$– intensity of heat flux with three components, volume, η—viscosity, *p*—pressure, α—degree of curing. Reducing the system of equations by mutual substitutions, we finally get a set of six equations with six unknowns: **v**—velocity vector, *T*—temperature, *p*—pressure, α—degree of curing.

## 3. Residual Stresses

### 3.1. Classification of Approaches Used

**Figure 2:**
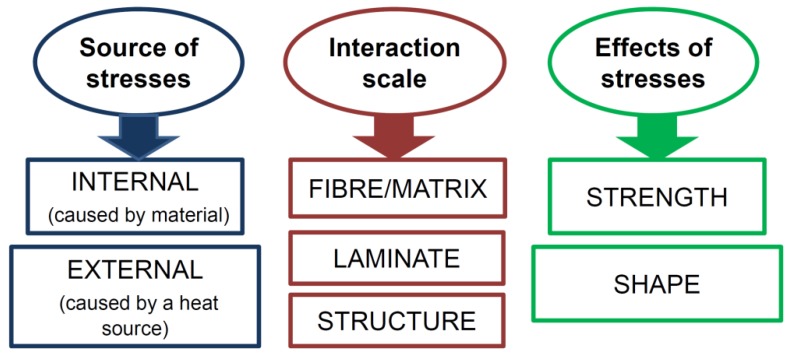
Possible levels of residual stress analysis.

Residual stresses and their influence on the structure of the composite material can be considered at various levels [8,12,63,64,65,66,67,68,69], i.e., the microscale (fiber/matrix), at the scale of the individual layer (laminate) or at the macroscale (structure)—Figure 2. At the level of the microstructure, the influence of residual stresses is usually taken into account by introducing a safety coefficient in the values determining allowable stresses and strength for composite materials. Some work in this field is carried out by Caiazzo et al. [70].

Most often, the residual stress induced by the resin curing process is calculated using the finite element method and/or the homogenization theory. This is done at the level of the individual layer, the laminate or the entire structure. Most of these works, however, concern the analysis of the resin curing process itself (without the analysis of filling molds by a liquid resin)—see Figure 1.

During and/or after the manufacturing process, the development of free strains and the mismatch of these strains between different components lead to the formation of residual stresses. In general, the free strains are divided into three categories: strains due to thermal changes (i.e., thermal expansion/shrinkage), strains due to phase changes of the matrix (i.e., cross-linking or crystallization) and strains due to moisture absorption, i.e.,:

(19) $${\tilde{\varepsilon }}_{j}^{free}(t)=\gamma_{j}(\alpha ,T,M)\Delta T(t)+\beta_{j}(\alpha ,T,M)\Delta M(t)+\varphi_{j}(\alpha ,T,M)\Delta \alpha (t),$$

where: *j*—the strain component, $\gamma_{j}$—the coefficients of thermal expansion, $\beta_{j}$—the coefficient of moisture expansion, $\varphi_{j}$—the coefficient of cure expansion, *M*—moisture, Δ—increments in the real physical time *t*. In the further part of the work, the influence of stresses/strains due to moisture absorption will be neglected.

**Figure 3:**
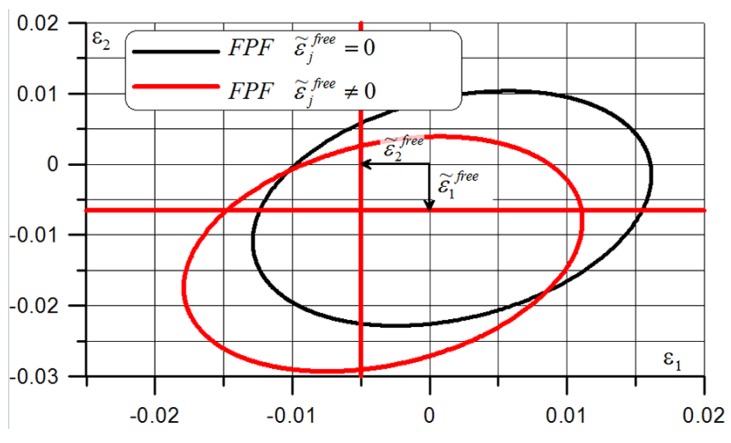
The effects of curing shrinkage (the Tsai-Wu criterion).

**Figure 4:**
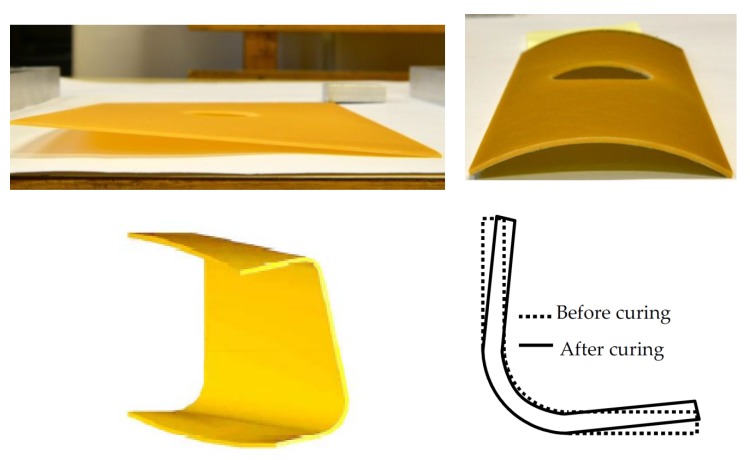
The effects of residual stresses -lateral torsional buckling of laminated structures (holes are drilled after manufacturing).

Internal heat sources generate residual stresses at the micro-scale level, and external ones at the macro level; they are applied on the edges of the structure. Manufacturing-induced residual stresses of polymer–matrix composites reduce the tensile load at which first ply failure occurs [71]—see Figure 3. Thermomechanical treatments offer the potential to change these residual stresses, but their application is hindered because the shape stability of composite material components is limited at treatment temperatures, which must be above the glass transition temperature of the matrix. The fundamental effect of residual stresses is observed in the form of the reduction of structural strength (see Figure 3) and as the change in the shape of the structure (lateral torsional buckling)—Figure 4.

In this work, we will analyze the effect of residual stresses at the laminate level taking into account the influence of external heat sources.

Theoretical and numerical modeling of thermal residual stresses was investigated in Refs [72,73,74,75,76,77,78]. Several techniques have been used to predict the spring-in of curved and angle sections ranging from simple analytical models [79,80] to laminate plate theory and finite element based models [40,47,59,60,81,82,83,84].

### 3.2. Viscoelastic Model

Using the classical linear viscoelastic approach (see e.g., Kim et al. [85]), the stress components can be evaluated from the following relation:

(20) $$\sigma_{i}(t)=\int_{0}^{t}Q_{ij}(\alpha_{c},\xi -\xi \prime )\frac{\partial }{\partial \tau }\left[\varepsilon_{j}(\tau )-{\tilde{\varepsilon }}_{j}(\tau )\right]d\tau ,$$

where *Q_ij_* denotes the components of the stiffness matrix. For the given value of the cure parameter α_c_ [86], it is assumed that:

(21a,b) $$\xi =\int_{0}^{t}\chi \left[\alpha_{c},T(s)\right]ds,\;\xi \prime =\int_{0}^{\tau }\chi \left[\alpha_{c},T(s)\right]ds,$$

The mechanical properties of the composite material change as the curing progresses. In particular, the transverse compliance, *S*_22_(*t*) = [*Q*_22_(*t*)]^−1^, undergoes a substantial change with time during cure [87]. This behavior can be modeled by a power law of the form:

(22) $$S_{22}(\alpha ,t)=S_{22i}(\alpha )f(t)+D(\alpha ){\left[t\chi_{T}(\alpha ,T)\right]}^{q(\alpha )},$$

where:

(23a,b,c) $$D(\alpha )=D_{i}+(D_{f}-D_{i})\alpha ,\;q(\alpha )=q_{i}+(q_{f}-q_{i})\alpha ,\;\chi_{T}(\alpha ,T)=\chi (\alpha ,T,M)$$

and *f* is a material dependent function chosen to agree with the experimental results [59,60], *D*—the transverse creep coefficient, α_T_—the shift factor and *q* is the transverse creep exponent. Assuming Young’s modulus varies with the cure parameter α, the form of the cure parameter variations with time, one can obtain the resultant stresses for the given laminate stacking sequences and their variations with time.

For a plane stress problems, the elements of the stiffness matrix [***Q***] can be expressed by the elements of the compliance matrix [***S***] as follows:

(24) $${\hat{Q}}_{11}(\alpha ,t)=\frac{S_{22}(\alpha ,t)}{S_{11}(\alpha )S_{22}(\alpha ,t)-S_{12}^{2}(\alpha )},$$

(25) $${\hat{Q}}_{22}(\alpha ,t)=\frac{S_{11}(\alpha )}{S_{11}(\alpha )S_{22}(\alpha ,t)-S_{12}^{2}(\alpha )},$$

(26) $${\hat{Q}}_{12}(\alpha ,t)=\frac{-S_{12}(\alpha )}{S_{11}(\alpha )S_{22}(\alpha ,t)-S_{12}^{2}(\alpha )},$$

where:

(27a,b,c) $$S_{11}(\alpha )=\frac{1}{{\hat{E}}_{11}(\alpha )},\;S_{12}(\alpha )=-\frac{{\hat{v}}_{12}(\alpha )}{{\hat{E}}_{11}(\alpha )},\;S_{22i}(\alpha )=\frac{1}{{\hat{E}}_{22i}(\alpha )},\;$$

The variations of Young’s transversal modulus (in the direction perpendicular to the fiber) can be written in the following way:

(28) $$\begin{array}{ll} {\hat{E}}_{22i}(\alpha )=E_{22} & 0\leq \alpha \leq \alpha^{*}\\ {\hat{E}}_{22i}(\alpha )=a_{0}+a_{1}\alpha +a_{2}\alpha^{2} & \alpha^{*}\leq \alpha \end{array}$$

where: ${\hat{E}}_{22i}$ is the initial transverse modulus, *E*_22_ is the transverse modulus for the uncured material, α* is the degree of curing for the initial modulus and *a*_0_, *a*_1_ and *a*_2_ are the parameters of the model.

Young’s modulus parallel to fibers and Poisson’s ratio are usually written as follows:

(29) $${\hat{E}}_{11}(\alpha )={\hat{E}}_{11i}+({\hat{E}}_{11f}-{\hat{E}}_{11i})\alpha ,$$

(30) $${\hat{v}}_{12}(\alpha )={\hat{v}}_{12i}+({\hat{v}}_{12f}-{\hat{v}}_{12i})\alpha ,$$

In Equations (24)–(30) the symbol “^” was introduced above the symbols to distinguish the quantities referring to the description of viscoelastic and elastic phenomena. It should be emphasized that Equations (24)–(28) are a generalization of analogous compounds for viscoelastic models. The physical meaning of the possibility of using such compounds is explained in detail in Pipes et al. [88]. A review of the applied models of viscoelastic bodies for the description of fiber composites is presented by Sun [89]. Sobotka [90] showed an overview of the rheological models used to describe the deformation of orthotropic plates and shells. Wilson [91] analyzed the effect of considering viscoelastic deformations on buckling of rods and plates. He showed that this reduces the critical force. Similar effects were noted for shell structures—see Rikards and Teters [92]. Problems of viscoelastic deformations in polymer mechanics are also discussed in Wilczyński’s monograph [93].

### 3.3. Mechanical 2D Model

Using the classical 2D relations for elastic thin-walled laminates, we can calculate the stresses as follows (compare with Equation (19)):

(31) $$\sigma_{i}=Q_{ij}\left(\varepsilon_{j}-{\tilde{\varepsilon }}_{j}^{free}\right),\;i,j=1,2,6,$$

Strains are assumed to change linearly through the thickness:

(32) $$\varepsilon_{j}=\varepsilon_{j}^{0}+z\kappa_{j},$$

where $\varepsilon_{j}^{0}$ is the vector of mid-plane strains and $\kappa_{j}$ is the vector of curvature components of the laminate. Defining in the classical manner the in-plane force resultants and moments resultants:

(33a,b) $$N_{i}=\int_{-t/2}^{t/2}\sigma_{i}dz,\;M_{i}=\int_{-t/2}^{t/2}\sigma_{i}zdz,\;i=1,2,6,$$

and writing the Equations (5) and (6) in the incremental form we obtain finally the values of mid-plane strain increments $d\varepsilon_{j}^{0}$:

(34) $$\sum_{k=1}^{NL}Q_{ij}^{k}t_{k}d\varepsilon_{j}^{0}-\left(\sum_{k=1}^{NL}Q_{ij}^{k}t_{k}d{\tilde{\varepsilon }}_{j}^{0free}\right)=0,$$

the positions of the neutral axis *z_b_*:

(35) $$\sum_{k=1}^{NL}Q_{ij}^{k}\left(t_{k}-z_{b}\right)\delta_{jr}=0,$$

and the increments of the curvature $d\kappa_{j}$:

(36) $$\sum_{k=1}^{NL}\;\int_{z_{k-1}}^{z_{k}}dzQ_{ij}^{k}\left[d\varepsilon_{j}^{0}+\left(z-z_{b}\right)d\kappa_{j}-d{\tilde{\varepsilon }}_{j}^{0free}\right]=0,$$

The above relations were derived under the assumption that the mold is not subjected to any constraints. However, the distributions of the free strains with respect to the temperature and the cure parameter should be known in advance from experiments.

If the vacuum bag is attached to the solid mold, both mid-plane strains and the curvature are constrained during cure and warpage is prevented. Therefore, under the assumption $d\varepsilon_{j}^{0}-d\kappa_{j}=0$ we can calculate the incremental force and moment due to the incremental cure shrinkage:

(37) $$dN^{mech}=-\sum_{k=1}^{NL}\;\int_{z_{k-1}}^{z_{k}}Q_{ij}^{k}d{\tilde{\varepsilon }}_{j}^{0free}\left(\alpha ,T\right)dz,$$

(38) $$dM^{mech}=-\sum_{k=1}^{NL}\;\int_{z_{k-1}}^{z_{k}}Q_{ij}^{k}d{\tilde{\varepsilon }}_{j}^{0free}\left(\alpha ,T\right)zdz,$$

At the end of curing, the total force and moment can be found by the integration over the total value of the cure parameter α and the temperature *T*.

### 3.4. Residual Stresses—Numerical Example

**Figure 5:**
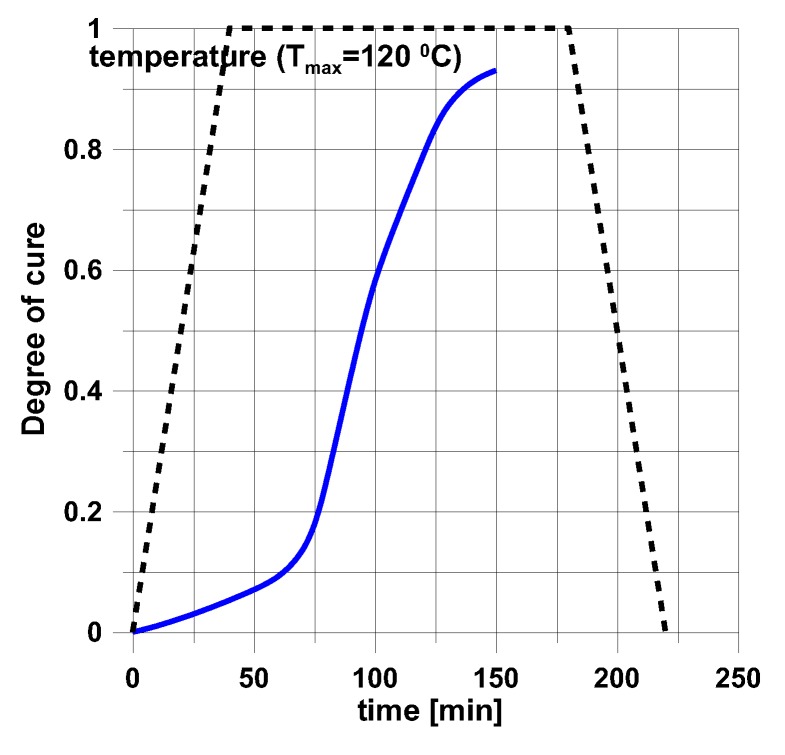
Variations of temperature and curing during the manufacturing of structures.

In the above presented models, residual stresses can be derived knowing the variations of the cure parameter α with time and temperature for the strictly specified type of a resin. For instance, in the mechanical model (see Section 3.3) the analysis of the hardening process is based on the diagram drawn below (Figure 5). The presented variations of temperature and cure parameter concern the production of layered cylindrical shells and plates with circular holes (see Figure 4). The structures were made of 8 layers with layers orientation ±45° using the autoclave technique. The cure parameter was calculated using the Kamal and Sourour model—Equation (13). The obtained results from the analytical solutions were compared with FE solutions and deflections of the real structures—Table 3.

The presented above examples refer to the simplest heating process in the autoclave corresponding to the trapeze form of the temperature-time profile – Figure 5.

Using the mechanical model, it is possible to compute the residual stresses. Now, the analysis is conducted for composite plates with cross-ply configuration 0°/90° taking into account two resin curing models:

Model 1—Kamal and Sourour—Equation (13) (k_1_ > 0),

Model 2—Bogetti and Gillespie—Equation (15) (k_1_ = 0).

**Figure 6:**
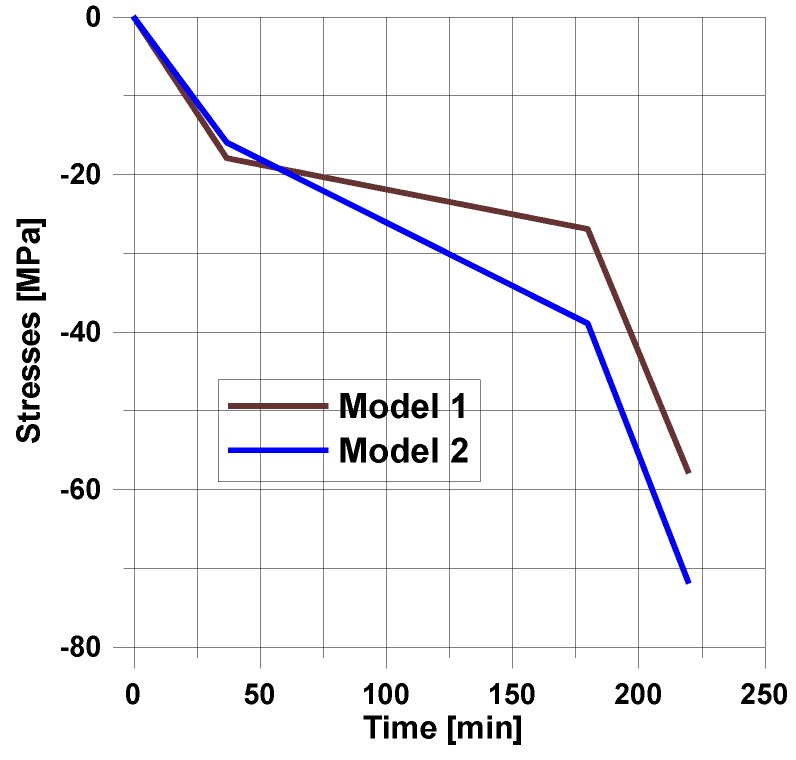
Distributions of residual stresses for the simplest heating and cooling process (see Figure 5).

For the assumed form of the temperature profile and the degree of cure distribution, the plots of the residual stresses are shown in Figure 6.

The final values of residual stresses are strongly affected by the prescribed curing model.

## 4. Optimization Problems

### 4.1. General Remarks

**Figure 7:**
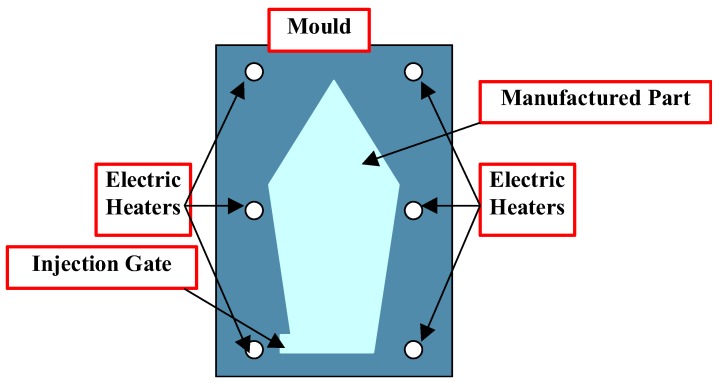
2-D cross-section of the mould with manufactured part.

Both technological processes discussed herein, i.e., the RIM and the RTM processes are conducted in a similar way presented in Figure 7.

The initially heated resin mixture is injected into the mould through the inlet gate— the number of the inlet gates may be greater than one. In the part of the mould occupied by the manufactured part, a porous media may exist that determines unidirectional, 2-D or 3-D systems of fibres in the case of the RTM process and one or more obstacles in the macro scale (or even none of them) in the case of the RIM process. In the mathematical or numerical sense, the difference between two processes results in the appearance of the additional set of equations (the Darcy flow rule—Equation (17)) for the RTM process whereas for the RIM process, the additional part inserted into the space occupied by the manufactured part in Figure 7 leads to the appearance of additional boundary conditions at the boundaries of obstacles. Then, the whole mould is heated to the prescribed temperature and then consolidated and cooled. The temperature in the mould is controlled by a finite set of electric heaters running around the mould in 3-D space.

In engineering practice, the final quality of the product and residual stresses obtained in the curing process are dependant on the variety of factors and each of them may be treated as the design variable in the optimisation problems. In general, they may be classified in the following manner:

Resin mixture:

- Type of the resin (polyester, epoxy etc.),
- Types of fillers and hardeners,
- Weight fractions of components,
- Relation between viscosity, time, temperature and degree of curing,
- Mold (Figure 7):
- Geometry,
- Positions, number and type (point or line) of the inlet gates,

Specific parameters of the technological process (Figure 7)

- Velocity of the resin mixture at the input gate,
- Pressure at the input gate,
- Initial temperature of the resin mixture,
- Application of vacuum (or not),
- Total number of products,
- Control of parameters during the process.

They are not independent parameters since everything depends on the existing equipment and the engineering realization of technological process.

**Figure 8:**
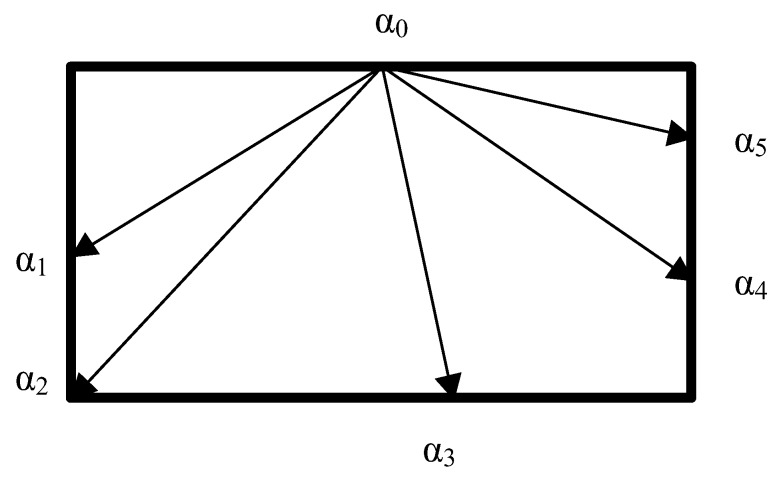
Schematic distribution of the degree of curing α.

However, the resin mixture flow may be blocked by the partially cured resin, i.e., the resin having (e.g., according to the commonly accepted assumption) a degree of cure α greater than 65%. In the optimisation problem, to eliminate such an inconvenience during the curing process, one may control the spatial distribution of the degree of cure α inside the mold at each time step. Strictly speaking, we intend to build the optimal spatial distribution of the α parameter in such a way that the degree of curing is a decreasing function in an arbitrarily chosen direction *s*, i.e.,:

(39) $$\frac{d\alpha }{ds}<0,$$

for an arbitrary moment of curing time *t*—see Figure 8.

The parameter *s* in Equation (39) denotes the vector joining two points P_0_ of the highest degree of curing and P_l_ where the curing parameter is measured. In general, that relation is valid for both 2-D and 3-D problems, i.e., for an arbitrary pair of points inside the mold.

**Figure 9:**
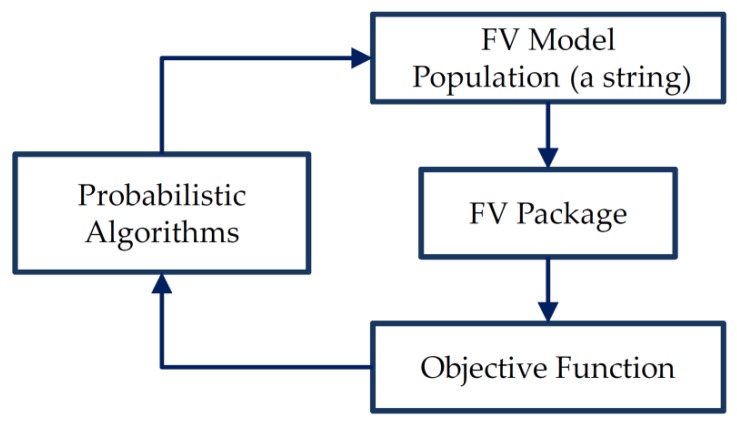
Schematic overview of the optimization process.

The detailed optimization analysis can be carried out with the use of the numerical analysis in the form shown in Figure 9.

In Figure 9 the term “probabilistic algorithms” denotes both genetic algorithms and evolutionary algorithms that may be used in the optimization problems. The detailed description of those procedures (not repeated herein) is discussed in detail by Muc et al. [94,95,96,97].

### 4.2. Optimization Examples

#### 4.2.1. RIM Process—Optimal Sequence of Hardening

It is obvious that the heating of the mould in the room or very low temperatures can reduce significantly or even eliminate the problems mentioned in the previous section. On the other hand, the quality of the product and its strength increase significantly for the curing process at high temperatures. Therefore, it is necessary to conduct the heating process at high temperatures but analysing both spatial as well as time distributions of the degree of cure *α* and these effects should be taken into account in the objective. Finally, the objectives of our optimisation problem can be formulated in the following way:

To maximize:

(40) $$F=\sum_{i=1}^{I_{h}}\frac{\int_{0}^{t_{k}}\alpha_{Pi}}{100\times t_{k}}-\sum_{t_{i}=1}^{N_{t}}A\times pen$$

where: *I_h_*—the total number of the control points, *t_k_*—the total time of the curing process, *α_Pi_*—the degree of curing at the control point *P_i_*, *t_i_*—the i-th time step, *N_t_*—the total number of time steps in the numerical simulation, *pen*—the penalty coefficient, *A*—the value computed from the formulae:

(41) $$A=\{\begin{array}{c} \frac{d\alpha }{ds_{P}}\;\mathrm{if}\;\frac{d\alpha }{ds_{P}}>0\;\\ 0\;\mathrm{if}\;\frac{d\alpha }{ds_{P}}>0 \end{array}$$

**Figure 10:**
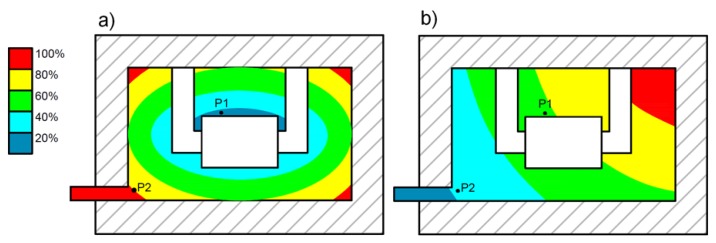
Comparison of distribution of degree of cure during curing process: (**a**) incorrect, (**b**) optimal.

The presented methodology was applied to the optimization of the reactive injection molding process (RIM) of the structure presented in Figure 10a,b. The main aims of the analysis were to optimize the degree of cure and the total curing time—Equation (40). Both parameters can be treated as the main effectiveness measures of the technological RIMP. Special attention was focused on the quality of the product in surroundings of the corners within molds. Non-optimal curing process (Figure 10a) may result in defects appearing (macroscopic and microscopic voids) in the final product. Influences of different parameters were studied in the optimization process and finally the temperatures of the heat sources were selected as the design variables. The calculations were made using Fluent software and with the application of the genetic algorithms. In the numerical analysis of the non-isothermal RIM the following phenomena were studied:

1. The behavior of the resin (fluid) during the injection (Navier-Stokes relations),
2. Heat transfer analysis (Fourier law),
3. Curing and rheology (including viscosity modeling—Castro-Macosko model and curing kinetics – Kamal and Sourour model) during the gelation.

Generally, macroscopic defects are caused by large air pockets, which can be blocked inside the mold cavity or stopped resin flow caused by the partially cured resin. This problem can be observed in Figure 10a, in which the curing process begins around the inlet gate (P2). Such an incorrect curing process leads to the appearance of the air bubbles. In order to prevent such phenomena during the technological process, the distribution and change of the degree of cure α of the whole structure at each moment of time should be controlled.

**Figure 11:**
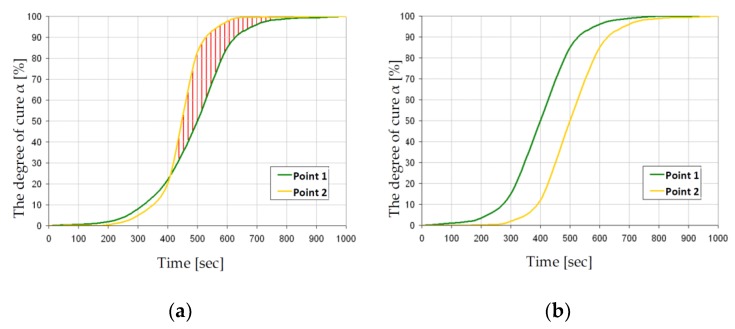
Variations of the degree of cure for different initial and boundary conditions of curing process: (**a**) incorrect curing process, (**b**) optimal curing process.

For a particular technological process, the curing process at two points (P1 and P2; where P1 – point in which curing should occur earlier than in point P2) can take the form shown in Figure 11 and it is not characterized by a single curve in the form presented in Figure 5. The area between curves, describing the curing process in time, for any two points of a structure can be treated as a measure of correctness of the curing process (Figure 11). If the curing curve for P1 lies above the curve for point P2, then the curing process has the correct manner (Figure 11b). In the otherwise case or in a case in which curves cross each other or have common points, the curing process is incorrect and may lead to the deterioration of the product quality (Figure 11a).

#### 4.2.2. RTM Process—Optimal Temperature Profile

In the RTM process, due to a variety of chemical exothermal reactions and curing procedures, residual stresses of non-mechanical origin can arise and finally may lead to the reduction of the load carrying capacity of the structure. Therefore the fundamental optimization problem takes the following form:

To minimize the residual stresses varying the time interval during the curing procedure:

*Min σ(t)* (42)

For the prescribed temperature variations (Figure 5), the distributions of residual stresses are demonstrated in Figure 6.

**Figure 12:**
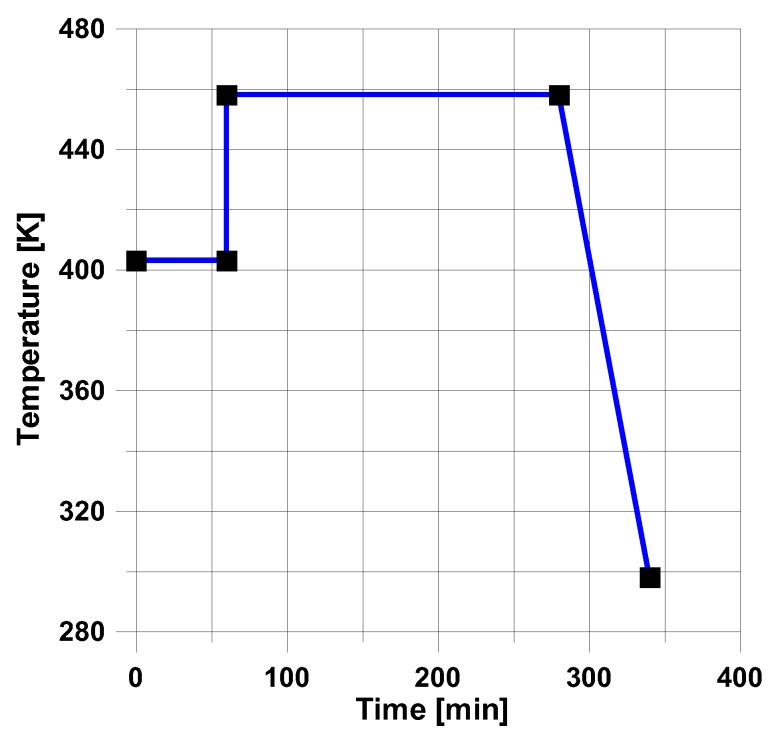
Definitions of design variables.

Now, it is assumed that the design variables *s_k_* may vary in time but in a specific, prescribed way, i.e.,:

(43) $$s_{k}(x_{i},t)=s_{k}(t_{})\begin{array}{cc} , & t\in [t_{p1},t_{p2}] \end{array}$$

where *k* denotes the total number of design variables, whereas *p1* and *p2* mean the number of time intervals in the assumed heating process—see Figure 12.

**Figure 13:**
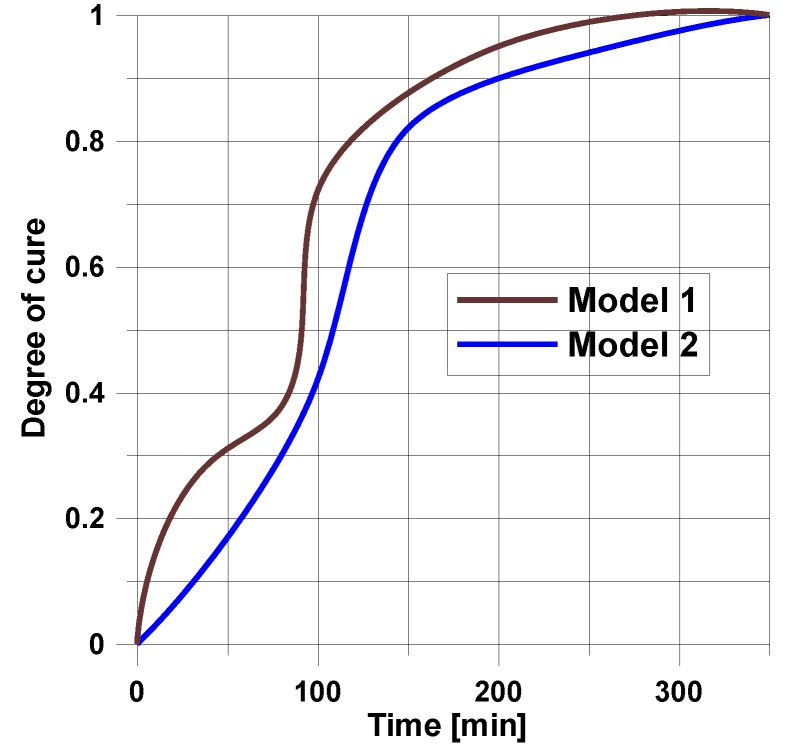
Variations of the degree of curing with time for the optimal heating/cooling process shown in Figure 12.

**Figure 14:**
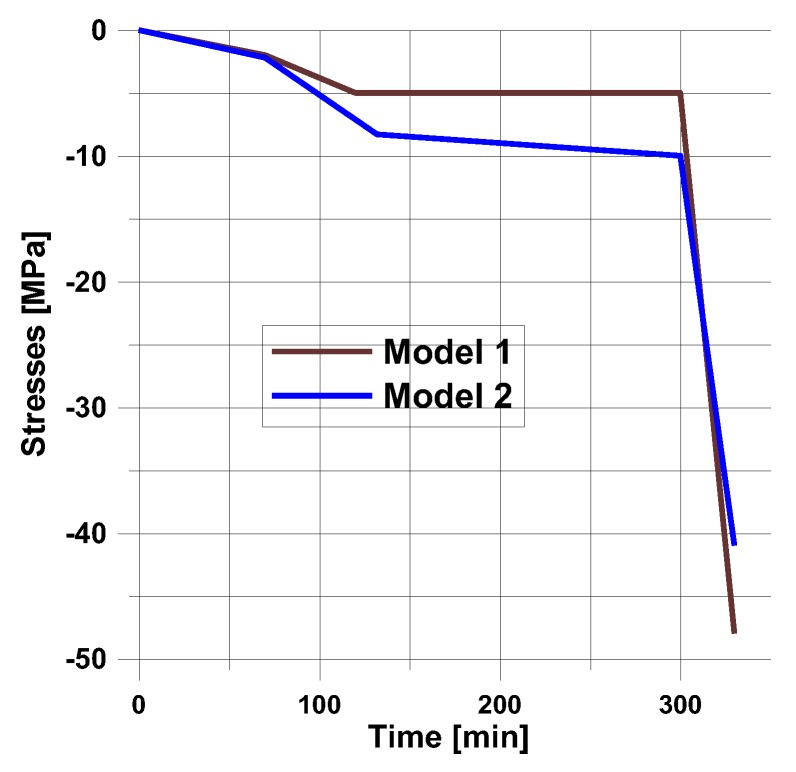
Distributions of residual stresses for the optimal heating and cooling process presented in Figure 12.

In the presented case, the total number of ten design variables ((*t_1_*,*T_1_*), (*t_2_*,*T_2_*), (*t_3_*,*T_3_*), (*t_4_*,*T_4_*), (*t_5_*,*T_5_*)) is reduced to only seven (see Figure 12). The analysis is conducted for cross-ply symmetric laminates [0_4_,90_4_]_S_ with the use of the viscoelastic model (the Section 3.2). Similarly, as previously, model 1 is described by the Kamal and Sourour relations, and model 2 by the Bogetti and Gillespie equation. The results of computations are plotted in Figure 13 and Figure 14.

## 5. Concluding Remarks

In the present paper, the possibility of the conjunction of genetic and evolutionary algorithms with the FV Fluent package in application to the optimization of the Reactive Injection Molding (RIM) and the Resin Transfer Molding (RTM) technological processes is presented and discussed. In the first part, a complete set of governing equations characterizing the thermosetting resin hardening process is written. Special attention is focused on the definition of design variables that can be used in the optimization problems for the above technological processes.

Among different variants of optimal designs, two 2-D problems are solved: (1) the optimal sequence of hardening and (2) the optimization of heating/cooling process and their influence on the values of residual stresses. The analysis conducted enables a better understanding of the behavior of the resulting residual stresses to changes in the cure cycle. It is concluded that by choosing these gradients in an optimum manner, the residual stresses can be reduced substantially.

The numerical results demonstrate evidently the strong influence of governing relations on the optimal solutions.

## Figures and Tables

**Table 1:** Validation of particular reaction kinetics models for composite materials and epoxy resin systems.

Material System	Reaction Kinetics Models	References
CFRP	1.Kamal–Sourour (13) & Arrhenius (14) 2. Bogetti–Gillespie (15) 3. Kempner et al. (16) 4. Kamal–Sourour (13) & Bailleul [27]	[38,39,40,41,42,43,44]
GFRP	1.Kamal–Sourour (13) & Arrhenius (14) 2. Bogetti–Gillespie (15)	[45,46,47,48]
Epoxy resin systems	1. Kamal- Sourour (13) & Arrhenius (14) 2. Kamal–Sourour (13) & Rabinowitch [28] 3. n–th order (10) 4. n–th order autocatalytic (12)	[49,50,51]
Fast cure epoxy	1.Model for non-isothermal curing based on the Kiuna approach [29] 2. Iso–conversional methods [30] 3. Model based on the Ruiz et al. approach [31,32]	[30,32,37]

**Table 2:** Validation of particular reaction kinetics models for selected fabrication processes of fiber composites.

Process	Reaction Kinetics Models	References
LSR	Kamal–Sourour (13) & Arrhenius (14)	[52]
RFI	Kamal–Sourour (13) & Arrhenius (14)	[53]
RTM	1. Kamal–Sourour (13) & Arrhenius (14) 2. Iso–conversional methods [30] 3. Ruiz–Trochu [31,33] 4. Extended Bogetti–Gillespie [34] 5. Kamal–Sourour (13) and Bailleul [27] 6. Prout–Tompkins autocatalytic (12)	[30,31,33,38,44,51,54]
C-RTM	Model based on the Ruiz et al. approach [31,32]	[32]
RIM	Iso–conversional methods [30]	[30]
VARTM	Kamal–Souror (13) & Arrhenius (14)	[55,56]
REX	Iso–conversional methods [30]	[30]
Autoclaving	Karkanas–Partridge’s (modificated Kamal–Sourour) [35,36]	[57]
OOA	1. Extended Bogetti–Gillespie [34] 2. Model for non–isothermal curing based on the Kiuna approach [37]	[34,37]

**Table 3:** Comparison of the maximal deflection obtained using analytical and FE models with experimentation.

Method	Deflection [mm]	
	Plate; 8 layers ±45°	Cylindrical shell; 8 layers ±45°
Technological process	31	20
Kamal and Sourour model—Equation (13)	25.4	17.9
FE model	22.8	16.9

## Appendix A.

**Table A1:** Physical properties of the epoxy resin used in the numerical examples. The model input parameters and values based on the experimental study conducted by White and Hahn [58,59] are given below.

Parameter	Value	Parameter	Value
First dwell temperature	131 °C	Degree of curing when chemical shrinkage is complete	α_c_ = 0.81
Second dwell temperature	181 °C	Final transverse chemical shrinkage strain	*e_2_^cf^* = −0.029
Cure kinetics constant	*k_1_* = 1.98exp(-2770/*T*) s^−1^	Uncured transverse creep coefficient	*D_i_* = 2.72E-9
Cure kinetics constant	*m_1_* = 1.17-(1.74E-3)*T*	Fully cured transverse creep coefficient	*D_f_* = 2.72E-9
Cure kinetics constant	*n_1_* = 199-(0.415)*T*	Uncured transverse creep exponent	*q_i_* = 0.123
Cure kinetics constant	*k_2_* = 6550exp(-7040/*T*) s^−1^	Fully cured transverse creep exponent	*q_f_* = 0.24
Cure kinetics constant	*m_2_* = 0	Shift factor constants	*B_1_* = 6190 *B_2_* = 20.3
Cure kinetics constant	*n_2_* = 13.2-(0.025)*T*	Initial transverse modulus modeling coefficients	*a_0_* = −214 GPa *a_1_* = 451 GPa *a_2_* = −228 GPa
Cure kinetics constant	*k_3_* = 81.9exp(-5340/*T*) s^−1^	Uncured transverse modulus	*E** = 2 GPa
Cure kinetics constant	*m_3_* = 0	Curing at initial transverse modulus development	α* = 0.82
Cure kinetics constant	*n_3_* = 131-(0.558)*T*+ +(6E-4)*T*^2^	Uncured longitudinal modulus	*E_11i_* = 114 GPa
First break point as a function of dwell temperature *Tc2*	(3.44E-12)(10^0.22·*Tc2*^)	Fully cured longitudinal modulus	*E_11f_* = 183 GPa
Second break point	-25.7+(0.11)*Tc2+* -(1.15E-4)*Tc2*^2^	Uncured major Poisson’s ratio	*υ_12i_* = 0.4
Longitudinal thermal expansion coefficient	α_1_ = −0.3E-6 m/°C	Fully cured major Poisson’s ratio	*υ_12f_* = 0.31
Transverse thermal expansion coefficient	α_2_ = 30E-6 m/°C	Minor Poisson’s ratio	*υ_21_* = 0.35
Initial stress-free temperature	*T_0_* = 290 K	Final shear modulus	*G* = 15 GPa
Chemical strain coefficients	*β_1_* = 0.005 *β_2_* = −525E-5 *β_3_* = 1	Half thickness of laminate	*h* = 0.765 mm
